# Abdominal ultrasound (FAST) in hemodynamically stable children with blunt abdominal trauma: study protocol for a randomized controlled trial

**DOI:** 10.1186/s13063-025-09137-6

**Published:** 2025-12-12

**Authors:** James F. Holmes, Daniel J. Tancredi, Kenneth M. Kelley, Mark Griffiths, Delia L. Gold, Samuel H. F. Lam, Bethsabee Stone, Timothy Brenkert, Amia N. Andrade, Eric Hanson, Kriti Gwal, Aaron Kornblith, Shinjiro Hirose, Garth H. Utter, Nathan Kuppermann

**Affiliations:** 1 Department of Emergency Medicine, University of California, Davis School of Medicine, Sacramento, CA USA; 2 Department of Emergency Medicine and Pediatrics, University of California, Davis School of Medicine, Sacramento, CA USA; 3 Division of Pediatric Emergency Medicine, Emory University School of Medicine, Atlanta, GA USA; 4 Pediatric Emergency Department, School of Medicine, Nationwide Children’s Hospital, The Ohio State University, Columbus, OH USA; 5 Department of Pediatrics, Section of Emergency Medicine, School of Medicine, Children’s Hospital Colorado, University of Colorado, Denver, CO USA; 6 Children’s Medical Center of Dallas, UT Southwestern School of Medicine, Dallas, TX USA; 7 Division of Emergency Medicine, Cincinnati Children’s Hospital Medical Center, School of Medicine, University of Cincinnati, Cincinnati, OH USA; 8 Department of Radiology, Nemours Children’s Hospital, Orlando, FL USA; 9 Department of Emergency Medicine and Pediatrics, University of California San Francisco School of Medicine, San Francisco, CA USA; 10 Department of Surgery, University of California, Davis School of Medicine, Sacramento, CA USA; 11 Departments of Pediatrics and Emergency Medicine, School of Medicine and Health Sciences, George Washington University, Washington, D.C., USA

## Abstract

**Background:**

Hemorrhage from intra-abdominal injuries (IAI) is a leading cause of traumatic deaths in children. Concern over misdiagnosing IAIs has resulted in excessive use of abdominal computed tomography (CT). Despite its many benefits, CT presents risks to children most notably radiation-induced malignancies. Thus, we must safely limit abdominal CT evaluation to those at non-negligible risk. The focused assessment with sonography for trauma (FAST) examination uses abdominal ultrasonography to detect the presence of intraperitoneal fluid in injured patients and may decrease abdominal CT use in some children. Limited and conflicting data exists on the utility of the FAST examination in children. A large multicenter study is thus necessary to determine if the FAST examination should routinely be included in the diagnostic evaluation of injured children.

**Methods:**

This is a multicenter, randomized controlled clinical trial to assess the impact of the FAST examination on the initial evaluation of children with blunt abdominal trauma. Enrolled participants will be randomized 1:1 to the FAST examination plus routine care or routine care alone during their initial emergency department (ED) evaluation. The study will enroll 3194 (initial sample size) to 4346 (second sample size) children at six diverse sites. The primary outcomes are as follows: (1) The proportion of abdominal CT in the initial 24 h of care and (2) the proportion of missed or delayed diagnoses of IAIs. Secondary outcomes include (1) ED length of stay, (2) hospitalization proportion and length of hospital stay, (3) physician suspicion of IAI, (4) the proportion of abdominal CT use in the subgroup of children 0 to 3 years old, and (5) laparotomy proportion. Hospitalized participants will be followed through their stay, and guardians of those discharged from the ED will be contacted after 1 week to assess their status.

**Discussion:**

The study will determine if the FAST examination results in a safe reduction of CT use in injured children and will provide definitive evidence if the FAST examination should be routinely implemented in the initial evaluation of children with blunt abdominal trauma.

**Trial registration:**

ClinicalTrials.gov NCT05910567. Registered on May 9, 2023

**Supplementary Information:**

The online version contains supplementary material available at 10.1186/s13063-025-09137-6.

## Introduction

### {#6a Background and rationale}

Hemorrhage from intra-abdominal injuries (IAI) is a leading cause of traumatic deaths in children [1]. Therefore, several consensus panels have placed the management of injured children as a research priority [2–4]. Many children with IAIs have subtle symptoms, making the diagnosis difficult, and missed or delayed diagnoses increase morbidity [5–8]. Excessive use of computed tomography (CT), however, also poses important risks, especially ionizing radiation exposure. CT is accurate in diagnosing IAIs, decreases the level of clinical monitoring required, and is important for determining the need for intervention including surgical treatment [9, 10]. However, due to the risks of radiation-induced malignancies [11–21], the use of CT should be optimized. Point-of-care ultrasonography has been proposed as a method to optimize CT use [22].

The focused assessment with sonography for trauma (FAST) examination uses abdominal point-of-care ultrasonography to detect intraperitoneal fluid in injured patients [23, 24]. The use of the FAST examination evolved in injured adult patients. Two randomized controlled trials in adults demonstrated that an evaluation strategy incorporating the FAST examination improves several aspects of patient care, including safely decreasing abdominal CT use [25, 26]. There are compelling reasons to thoroughly evaluate injured children for IAIs; however, avoiding CT evaluation in those at low risk is also critical. The FAST examination is clinically indicated in hemodynamically unstable, injured children; it also may enhance the diagnostic evaluation and decrease the frequency of CT use in low-risk children. However, the data are limited on the utility of the FAST examination in stable pediatric trauma patients [27–31]. A large, multicenter, observational study suggests that the FAST examination safely decreases CT use in children at low risk for IAI [31]. However, a separate observational study suggests that “a negative FAST aids little in decision-making,” in injured children, questioning its routine use [27]. Furthermore, a single-center, randomized controlled trial of FAST in injured children at low (~5%) risk for IAI conducted by our group demonstrated no difference in the frequency of CT use in the FAST versus the non-FAST study arms [28]. That trial demonstrated, however, that physician suspicion of IAI significantly decreased after a negative FAST examination, but this post-FAST decrease in suspicion did not decrease CT use [28]. Finally, a 2021 meta-analysis suggests the FAST examination may decrease CT use but only in the lowest risk children [30]. The conflicting results from these studies strongly indicate the need for a multicenter clinical trial powered to answer this critical question definitively.

### {#7 Objectives}

The study’s primary objective is to determine the impact of the FAST examination on clinical care in hemodynamically stable children with blunt abdominal trauma.

Finally, as part of this study, we will attempt to identify patient, clinician, and system factors associated with obtaining abdominal CT scans in patients considered low risk for IAI by the clinician and who also have a negative FAST examination. This planned analysis will be conducted only on those patients in the FAST study arm. To obtain clinician factors, we will survey all eligible clinicians prior to their participation in the study. Specifics on this sub-aim are not described in this protocol manuscript.

### {#8 Trial design}

This is a multicenter randomized controlled, superiority trial.

## Methods: participants, interventions, and outcomes

### {#9 Study setting}

We will perform this study in the Pediatric Emergency Care Applied Research Network (PECARN), a national pediatric emergency care research network with a long history of successful multicenter studies, especially randomized controlled trials and studies in injured children. The six participating centers are geographically diverse with a heterogeneous patient population. Participating EDs include the following: the University of California, Davis (Sacramento, CA, USA), the Children’s Hospital Colorado (Denver, CO, USA), Cincinnati Children’s Hospital Medical Center (Cincinnati, OH, USA), Children’s Healthcare of Atlanta — Egleston Pediatric Hospital (Atlanta, GA, USA), Nationwide Children’s Hospital (Columbus, OH, USA), and the University of Texas Southwestern Medical Center (Dallas, TX, USA). All sites are urban, academic centers with dedicated pediatric EDs serving as level 1 pediatric trauma centers, including dedicated trauma teams composed of emergency medicine physicians, pediatric surgeons, and trauma surgeons.

### {#10 Eligibility criteria}

Children up until their 18th birthday will be assessed for eligibility as (1) this is the age range evaluated by most pediatric EDs and the age range in the single-center randomized trial [28], and (2) mechanisms and injury responses are different in those older than 18 years. Inclusion and exclusion criteria are designed to enroll a hemodynamically stable population with approximately 5% having IAI. This is because a prior study suggests that use of the FAST examination safely decreases abdominal CT use in those children considered to have a 1 to 10% risk of IAI [31]. Furthermore, a meta-analysis of the FAST examination in injured children suggests that CT may be avoided in low-risk children with normal FAST examinations; however, the FAST should not be used to rule out injury in children at higher risk [30]. Inclusion and exclusion criteria follow the same criteria used in the prior single-center randomized controlled trial of FAST in children in which 5.4% had IAIs and are presented in Table 1 [28].

**Table 1:** Inclusion and exclusion criteria

Inclusion criteria [28]:
• Blunt torso trauma from a significant mechanism of injury:
◦ Motor vehicle collision:> 60 mph, ejection, or rollover
◦ Automobile (>25 miles/hour) versus pedestrian/bicycle
◦ Falls greater than 20 feet in height
◦ Crush injury to the torso or assault to the abdomen
• Glasgow Coma Scale score of 9-14 with blunt torso trauma
• Blunt traumatic event with extremity paralysis or multiple long bone fractures, regardless of the mechanism
• History and physical examination suggestive of abdominal injury following blunt torso trauma of any mechanism
Exclusion criteria [28]: Patients will be excluded for:
• Age-adjusted hypotension
◦ Prehospital or initial emergency department age-based hypotension. [57]
• Initial Glasgow Coma Scale score of 8 or less
• Penetrating trauma: Patients who are victims of stab or gunshot wounds
• Traumatic injury occurring more than 24 hours prior to the time of presentation to the emergency department
• Transfer of the patient with an abdominal CT scan, FAST examination or laparotomy previously performed
• Patients with known disease processes resulting in intraperitoneal fluid including liver failure and the presence of ventriculoperitoneal shunts

Hemodynamically unstable injured children are excluded as immediate FAST is recommended [32, 33]. Thus, equipoise on FAST use does not exist in children who are hypotensive/severely injured after blunt abdominal trauma.

### {#26a Who will take informed consent?}

Informed consent of the guardians for follow-up via email, telephone, or texting will be obtained by trained research coordinators and clinicians who provide care at the ED visit. Provider enrollment without research coordinator help has been successful in many PECARN studies.

### {#26b Additional consent provisions for collection and use of participant data and biological specimens}

This trial does not involve the collection of biological specimens. Secondary analyses and manuscripts are anticipated from the data collected, including the use of ultrasound for identifying pneumothoraces in children (eFAST) and the use of machine learning to interpret FAST images.

## Interventions

### {#6b Explanation for the choice of comparators}

Due to the clinical equipoise around the use of FAST in stable injured children, this trial will compare the FAST examination plus routine care versus routine care alone.

### {#11a Intervention description}

Bedside FAST examinations will be performed by the ED physicians providing care to the participants randomized to the FAST arm. Only ED physicians credentialed in the FAST examination will participate in the study. The FAST examinations will be performed using a portable ultrasound machine with low-frequency curvilinear or phased array probes (appropriate for patient size). Credentialing will follow guidelines for competency in point-of-care ultrasound established by the American College of Emergency Physicians, including the following: (1) understanding indications, (2) ability to obtain adequate images, and (3) correctly interpreting these images [34].

The intervention follows the FAST protocol for the detection of intraperitoneal fluid. This protocol includes views of Morison’s pouch (right upper quadrant), splenorenal fossa (left upper quadrant), and long and short axis of the pelvis and the heart. Images of extended-FAST (lung views) will not be required, but whether they are obtained by the physician will be collected on the case report form. No attempts to image the solid organs are required for the protocol, as this is not routine in the FAST examination. Clinicians are asked to record the results of the FAST examinations on the ultrasound machine as soon as the images are obtained. When possible, these FAST examination images will be uploaded via secure medical center Wi-Fi interface and stored on an IT-managed hospital secure server. Deidentified images will be uploaded into the Research Electronic Data Capture (REDCap) database. In some cases, the images will not be able to be saved and will no longer be available for storage and future review.

**Fig. 1:**
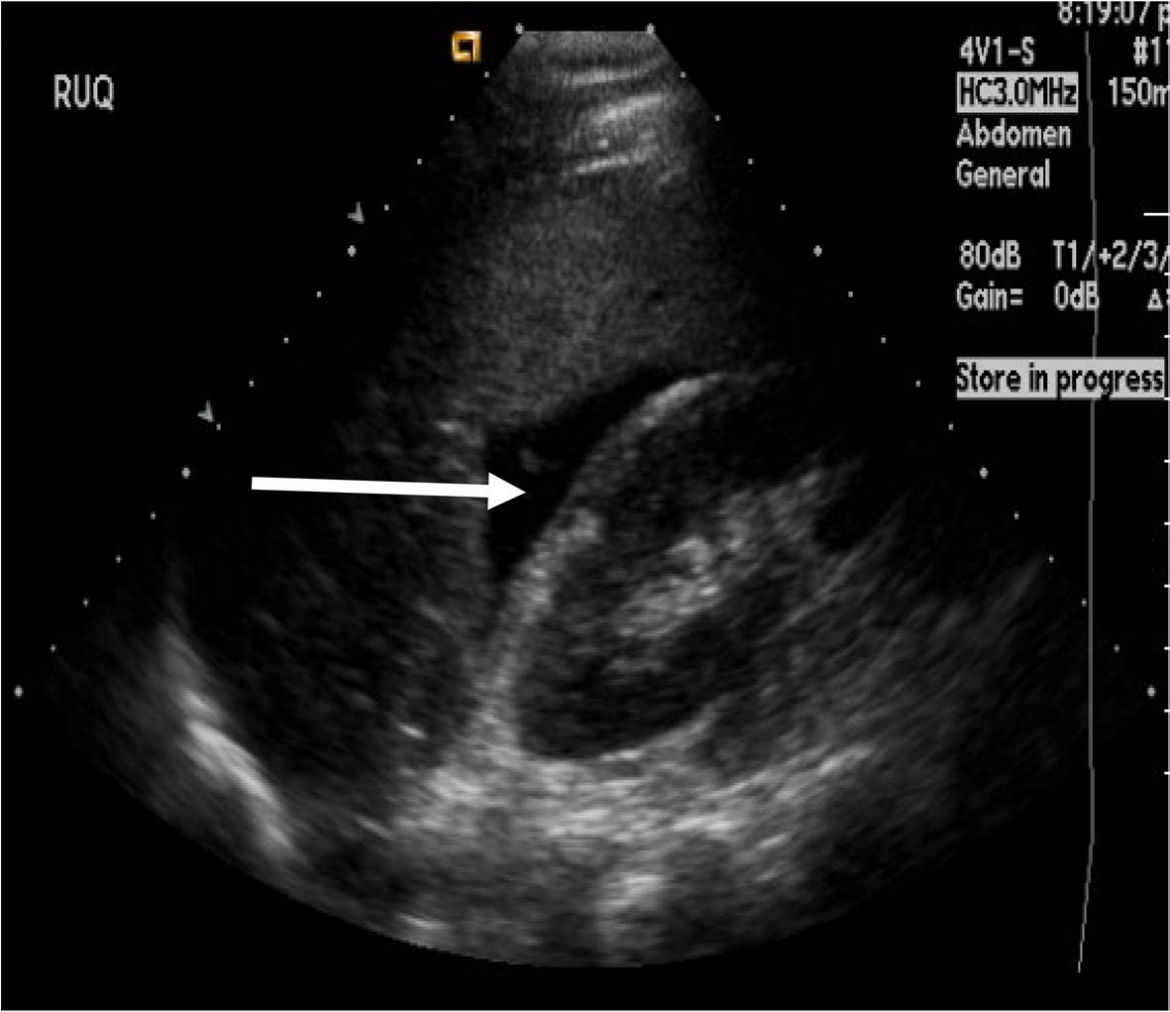
FAST image of the right upper quadrant view: The hypoechoic (black) area between the liver and renal parenchyma (arrow) is hemoperitoneum. This FAST is positive.

**Fig. 2:**
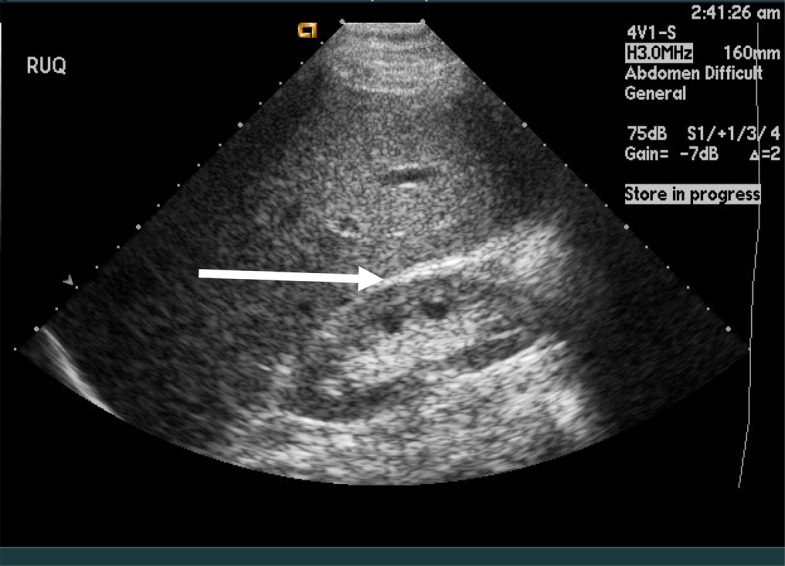
FAST image of the right upper quadrant view. No fluid is found between the liver and renal parenchyma (arrow). This FAST is negative

ED physicians (faculty or fellow physicians) caring for the participants will make bedside interpretations and record them onto case report forms. For analysis, FAST examinations will be classified as positive if any intraperitoneal fluid is identified (Fig 1.) or negative if no such fluid is noted (Fig. 2). The ED physician will also document the location of the fluid, if positive. We will not collect data on incidental findings or specify how treating physicians evaluate any findings that they may incidentally identify. Clinical action based on FAST findings will be at the discretion of the treating physicians.

All saved FAST examinations will also be presented for interpretation at each site per their quality improvement process to an expert ED ultrasonographer, unblinded to the initial interpretation. This person will be fellowship-trained in ED ultrasound and different from whom performs the study FAST examination. A separate study radiologist/ultrasonographer at the UC Davis Data Coordinating Center will review all FAST examinations in two circumstances: (1) ED physician interprets the FAST as negative for intraperitoneal fluid, and the site’s expert interprets it as positive, and (2) ED physician interprets the FAST as positive for intraperitoneal fluid, and the site’s expert interprets it as negative. If the UC Davis Data Coordinating Center expert agrees with the study site’s expert, the ED physician performing/interpreting the FAST examination will be informed that they misinterpreted it. The individual site leader will notify the ED physician of the discrepancy. This will not occur in real time.

### {#11b Criteria for discontinuing or modifying allocated interventions}

Based on perceived clinical necessity, the faculty physician caring for a patient in the non-FAST arm may perform a FAST examination. This would only be expected, however, in unusual cases such as the patient becoming hypotensive after randomization. This will be permitted by study protocol, as we cannot ethically interfere with patient care needs perceived by the faculty physician. We expect, however, that this will occur uncommonly (2.6% of cases in the single-center trial) [28].

### {#11c Strategies to improve adherence to interventions}

Adherence to randomization allocation is monitored monthly. Monthly compliance reports are sent to all participating sites, and compliance is discussed at each monthly call. Those sites whose allocation compliance falls below 95% have additional discussion and monitoring.

### {#11d Relevant concomitant care permitted or prohibited during the trial}

The study does not restrict care and interventions permitted during the trial.

### {#30 Provisions for posttrial care}

All participants will receive care as recommended by their care providers. No provisions for posttrial care beyond the standard care recommended are provided.

### {#12 Outcomes}

Primary outcomes will be measured and documented in both study arms. These outcome measures are the same as those used in the single-center randomized controlled trial. The primary outcomes are the abdominal CT proportion and the proportion of missed/delayed diagnosis of IAIs. The abdominal CT proportion will be calculated as the number of participants receiving abdominal CT scans in the initial 24 h divided by the number of participants in each arm (i.e., proportion of participants undergoing CT during their initial ED evaluation). Abdominal CT scans will be obtained at the discretion of the treating physicians in the ED. The other primary outcome is missed or delayed diagnosis of IAI. A missed diagnosis of IAI is defined as a diagnosis of IAI made after the patient is discharged from the hospital (if admitted) or from the ED (if not admitted to the hospital). A delayed diagnosis of IAI is defined as a diagnosis made after patient transfer from the ED to the hospital (including ward/intensive care unit bed). A delayed diagnosis may only be identified in a patient admitted to the hospital. Complications due to the missed/delayed diagnosis will be collected. We anticipate missed/delayed diagnoses to be rare but will nonetheless carefully assess for these as the FAST examination will require acceptance by clinicians that important injuries will not be missed if it is to be incorporated into practice.

Secondary outcome measures will also be collected and analyzed in both study arms. Although these outcomes are not the focus of the analysis, they will be gathered as they are important for assessing the utility of a pediatric trauma evaluation strategy using the FAST. Secondary outcomes include the following: (1) ED length of stay, (2) time to CT, (3) proportion of participants hospitalized and length of hospital stay, (4) proportion with abdominal CTs obtained in the subgroup of children 0 to 3 years old, (5) laparotomy proportion, and (6) physician suspicion of IAI.

ED length of stay is a continuous variable defined as the time (minutes) from ED arrival to ED disposition (ED discharge, hospital admission orders written, transfer to operating suite, or death). Abdominal CT time is defined as the start of the CT, and laparotomy time is defined as the time of entering the operating suite. Both of these are continuous variables and will be measured and compared as such. Time to CT or laparotomy will be measured from the time of ED arrival.

The hospitalization proportion is the number of participants hospitalized in each study arm divided by the number of participants in each arm (proportion admitted to the hospital from the ED). Participants will be considered hospitalized if they meet any of the following: (1) Admitted to observation status, (2) admitted to the intensive care unit or ward, and (3) transported from the ED to the operating suite/interventional radiology. We will also measure the length of stay for those hospitalized. This will be a continuous variable measured in hours from the time of ED arrival to discharge from the hospital or death in the hospital.

The proportion of abdominal CT use in children 0 to 3 years old is the number of abdominal CT scans performed in each study arm divided by the number of participants in each arm (proportion of participants undergoing an abdominal CT during their initial ED evaluation) among the population 0 to 3 years old. Laparotomy proportion is a binary indicator for whether the patient underwent a laparotomy or laparoscopy to identify/repair an IAI.

In those participants who undergo the FAST examination, we will also measure the impact of the FAST on the clinicians’ subsequent assessments of risk of IAI. In all participants, regardless of randomization, we will ask if the clinician believes the patient should have an abdominal CT scan and will measure clinician suspicion of IAI (classified by the clinician as < 1%, 1 to 5%, 6–10%, 11–50%, and over 50%). In those who are randomized to the FAST examination, the clinician will, after the FAST examination, again document if they believe the patient should have an abdominal CT and their suspicion of IAI (with the same categories). Finally, the clinician will, after performing the FAST examination, document if the FAST changed their suspicion for IAI or plans for patient evaluation or management.

### {#13 Participant timeline}

Hospitalized participants are followed through their hospitalization (up to 30 days from the initial ED visit) to identify outcomes. The guardians of participants discharged from the ED will be contacted (by telephone or text) at least 7 days from the initial ED visit to assess their status. We will make up to four attempts by telephone or text to contact the guardians over 3 months after the index ED visit, starting on day 7.

### {#14 Sample size}

The power of these analyses depends on the proportion of participants receiving abdominal CT in each stratum. The abdominal CT proportions in the prior randomized controlled trial using these entry criteria were 52.4% and 54.6% in the two arms [28]. In estimating sample size, we chose a baseline abdominal CT proportion of 52.5%. A minimum clinically important difference of 5% was specified for the abdominal CT proportion (from an expected proportion of 52.5%). A difference of 5% (number needed to receive the FAST examination = 20) was chosen because 74% of physicians (162/180 pediatric emergency medicine physicians surveyed responded at 18 PECARN centers) were willing to perform at least 20 FAST examinations to avoid one abdominal CT. This includes 35% of physicians who reported no limit on the number of FAST examinations they would be willing to perform to safely decrease abdominal CT use.

Sample size modifications are required to adjust for interim analyses, although the increase is slight for conservative alpha-spending functions. We initially specified that a sample size of 3194 was needed to detect a 5-percentage point difference (for 80% power) using a four-group sequential analysis design. It subsequently became apparent during the initial enrollment period that the baseline CT proportion was lower (34%) and enrollment was higher than planned. Thus, we calculated a second sample size to detect a 4% difference in CT proportions which would still be meaningful.

**Fig. 3:**
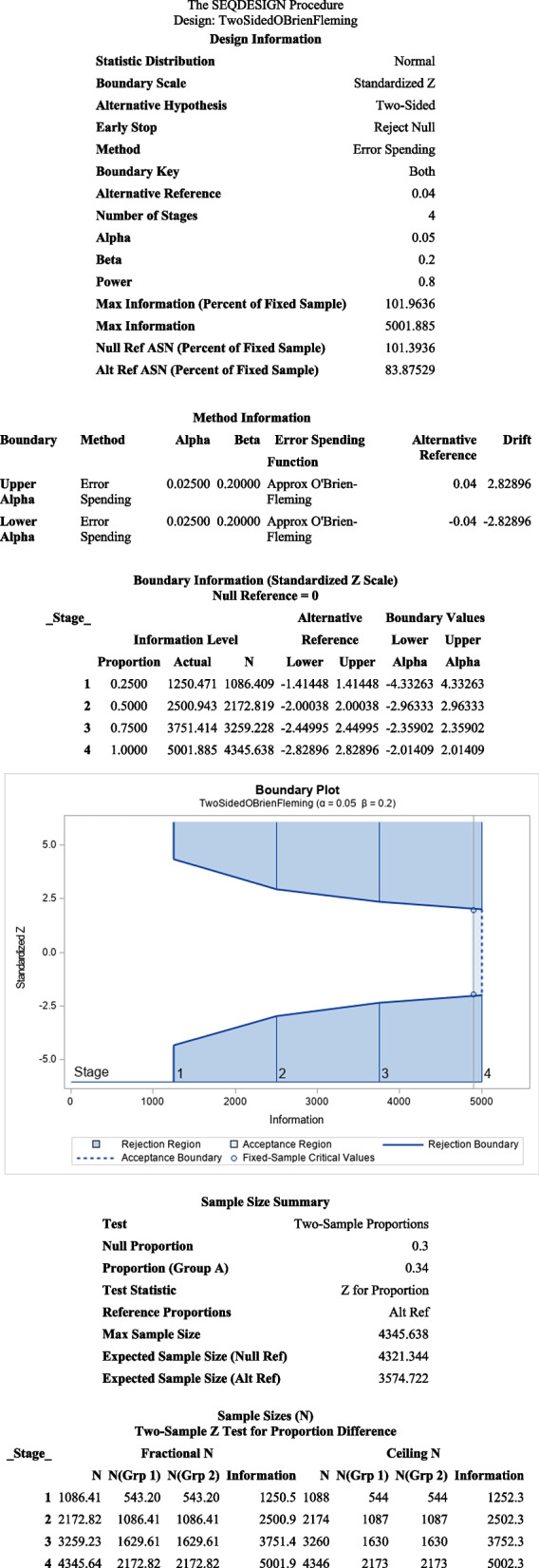
Sample Size Calculation in SAS

We subsequently specified a four-stage group sequential design (with three interim analyses and one final analysis) with possible early stopping only to reject the null hypothesis in Statistical Analysis System (SAS) PROC SEQDESIGN and determined that a sample size of 4346 would provide a power of at least 80% for detecting the more difficult-to-detect 4% difference between groups in the proportion of CT use, which is when one group has 30% CT usage and the other has 34% CT usage. To permit replication, Figure 3 displays the SAS output from the PROC SEQDESIGN used to produce the sample size targets for the study design in our protocol.

For those participants in the 0- to 3-year-old cohort, a 10% difference in abdominal CT proportions is clinically important. A 12% difference was found between arms in the single-center trial, but the sample size was too low to demonstrate statistical significance [28]. Assuming a baseline CT of 52.5% in all strata, a sample size of 796 participants in this age cohort would provide a power of at least 80% for detecting a 10% difference in the proportion of CT use between cohorts. Although a smaller difference may still be clinically meaningful, we are challenged by the feasibility of enrolling so many children in this age cohort.

We anticipate missed/delayed diagnoses of IAI to occur rarely. Among the 925 patients enrolled in our single-center FAST randomized controlled trial, only one missed/delayed diagnosis of IAI occurred (likely a false-positive CT and not a true missed IAI) [28]. Only 4 occurred in the PECARN observational study of 12,044 injured children [8]. Such outcomes are too infrequent to form a reliable basis for study sample size planning.

### {#15 Recruitment}

**Fig. 4:**
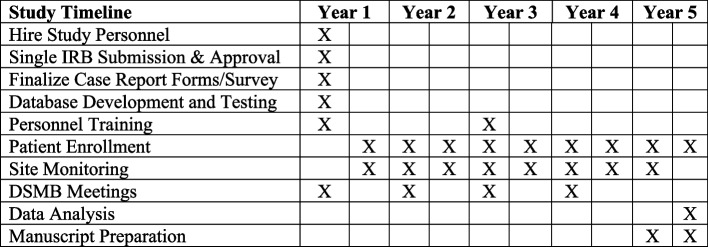
Study Timeline

The study will enroll eligible patients as they present to the ED. Figure 4 provides the timeline for the study.

## Assignment of interventions: allocation

### {#16a Sequence generation}

Patients meeting enrollment criteria will be randomized to the FAST or no-FAST arms using computer-generated randomly permuted blocks by enrollment order (investigators will be masked to the order). In addition, randomization will be stratified by site and age cohort. These actions will ensure appropriate randomization, and that confounding variables (either known or unknown) are likely to be equally distributed between study arms.

### {#16b Concealment mechanism}

To ensure allocation concealment, sealed, opaque envelopes were shipped from the UC Davis Data Coordinating Center to all study sites. These envelopes are (1) readily available in the ED, (2) reveal the randomization assignment stratified by age cohort (younger than 3 years or 3 to 18 years old), and (3) contain the appropriate case report form. The sequentially numbered, sealed opaque enrollment packets are located near the ultrasound machine and are always available to the providers once eligibility is determined. Once opened, these packets will reveal study arm allocation and provide all pertinent case report forms.

### {#16c Implementation}

The allocation sequence was generated by the study statistician. Participants will be enrolled by trained research coordinators and clinicians providing care. PECARN studies have previously successfully conducted studies where treating clinicians themselves enroll eligible patients.

## Assignment of interventions: blinding

### {#17a Who will be blinded}

Treating physicians will not be blinded to the results of the FAST examinations. As the study evaluates provider actions (i.e., obtaining an abdominal CT) based on the FAST results, it would be inappropriate to blind the clinicians providing care. It would also be unethical to do so, as FAST results could potentially reduce harm to participants. Participants, by definition, will also not be blinded to study arm, but any potential bias from their knowledge that the FAST was performed should be minimal. Assessment of all outcomes will be performed by trained research coordinators blinded to study arm. All statistical analyses will be performed with the statistician blinded to study arm. After the statistical analysis is performed and reviewed with the study team, all results will be unblinded.

### {#17b Procedure for unblinding if needed}

Emergency unblinding of the intervention is unnecessary for this study as the clinicians will know if they provided a FAST examination.

## Data collection and management

### {#18a Plans for assessment and collection of outcomes}

We will follow the enrollment and data collection procedures which were proven highly successful in the prior single-center randomized controlled trial [28] and learned over two decades in PECARN. At the time of the patient’s arrival, the faculty ED physician and/or research coordinator will immediately evaluate and determine patient eligibility. As per ED protocol, each enrolled patient will undergo a complete trauma history and physical examination by the responsible faculty physician, and findings will be documented by that faculty physician on a standardized case report form. Although no laboratory tests are required by protocol, we will record the results of the hemoglobin/hematocrit, liver transaminases, lipase, and urinalysis (for blood), if obtained. Emergency department data will be collected using standardized case report forms.

### {#18b Plans to promote participant retention and complete follow-up}

Guardians of enrolled participants discharged from the ED will be contacted by text/telephone beginning 1 week after discharge from the ED visit for up to 3 months. Evidence from prior studies indicated missed IAIs manifest within 48h of injury [8, 28]. There is no specific retention plan for this study.

### {#19 Data management}

Data is entered into a REDCap database created by the UC Davis Data Coordinating Center. Research coordinators manually enter data from case report forms or directly from the electronic medical record when pertinent (radiology reports, laboratory values, laparotomy reports). Saved FAST examinations will also be uploaded into REDCap as previously described. Data entered into REDCap will undergo multiple levels of quality control to minimize errors. Built-in field validation (numeric ranges, date formats, and required fields) will be enabled at the time of entry to prevent implausible values. In addition, branching logic and calculated fields will be used to trigger immediate alerts when data are inconsistent or fall outside expected ranges. Standard REDCap data quality rules and custom queries will be run on a routine basis to identify outliers, missing data, and discrepancies. Regular data quality reports will be generated, reviewed, and documented, and anomalies will be resolved in a traceable manner. Missing data fields will prompt automated queries in REDCap. All case report forms will be retained for 10 years.

### {#27 Confidentiality}

To ensure patient confidentiality, the paper case report forms will be kept in a secure location per each site’s policy. All data entered into the REDCap database will be deidentified. The database will additionally be password-protected. UC Davis provides effective firewall hardware, automatic network intrusion detection, and the expertise of dedicated security experts to ensure database security.

### {#33 Plans for collection, laboratory evaluation, and storage of biological specimens for genetic or molecular analysis in this trial/future use}

No biological specimens are being collected or stored as part of this trial.

## Statistical methods

### {#20a Statistical methods for primary and secondary outcomes}

The primary analysis will be based on the intention-to-treat principle and will include all randomized patients, who will be analyzed according to their randomized assignment. In addition, we will conduct analyses on the per-protocol population, the subset of the intention-to-treat population who received the randomly assigned intervention. Finally, we will analyze the as-treated population, which will include all randomized patients, who will be in groups defined by whether they did or did not receive the FAST examination.

Point estimates and confidence interval (CI) estimates of the effect size will be calculated using Zhao’s estimators for the probability that a randomly sampled observation from the FAST arm is at least as big as a randomly sampled observation from the non-FAST arm, holding stratum fixed, and an effect whose true value is 34% under the null hypothesis [35, 36]. The assumption that FAST versus non-FAST effects are homogenous across strata will be assessed by the rank-based approach of Akritas, Arnold, and Brunner [37].

The primary outcome is the proportion of abdominal CT use in each study arm, measured for each patient categorically (yes/no). We will use the Cochran-Mantel–Haenszel risk difference test to determine if differences in CT proportions exist between the two arms. The other primary outcome is the proportion of missed or delayed diagnoses of IAIs. The proportion of missed or delayed diagnoses of IAIs is anticipated to be very small [8, 28]. We will assess and report the important outcome of missed/delayed diagnoses for each study arm and compare their relative frequencies using Agresti-Min 95% CIs (with Agresti-Caffo intervals used as a backup in case of computational difficulties). Both interval types are recommended for small numerators [38]. Secondary outcomes include the following: (1) ED length of stay, (2) time to abdominal CT, (3) hospitalization proportion and length of hospital stay, (4) physician suspicion of IAI, and (5) laparotomy proportion.

All analyses will account for site, sex, and age stratum differences. Baseline demographic (including age and sex), clinical and laboratory data, and outcome measures will be compared between the two cohorts (FAST versus the no-FAST study arms) using appropriate bivariable statistical methods to assess for parity between arms for known confounding variables (e.g., mechanism of injury, PECARN Abdominal Prediction Rule variables). Ninety-five percent CIs will be calculated for all measures of interest. All tests will be based on two-tailed alternatives. *P*-values < 0.05 will be considered statistically significant and adjusted for interim analyses. Categorical data will be compared using Cochran-Mantel–Haenszel risk difference testing and estimation method for stratified samples, using the Miettinen-Nurminen (score) confidence limits available in SAS for the common risk difference. McNemar chi-squared test will be used to compare differences in proportions between clinician suspicion of intra-abdominal injury before and after the FAST examination. Clinician suspicion will additionally be collapsed and compared into those with less than 1% suspicion and those with 1% or greater suspicion of IAI.

All continuous data will be assessed using nonparametric statistical analysis procedures for stratified data, with hypothesis testing using the Wilcoxon-Mann–Whitney procedure on outcomes that have been rank-aligned by stratum using the Hodges-Lehman estimator, a more powerful alternative to the van Elteren test [39]. ED length of stay and time to abdominal CT are continuous variables measured in minutes. We will compare ED and hospitalization lengths of stay between participants in the two study arms using nonparametric methods (described above). Hospitalization and laparotomy proportions are categorical variables (yes/no) and will be compared using methods described above for categorical variables.

### {#21b Interim analysis}

We will perform three interim analyses. Interim monitoring for superiority of one approach over the other is appropriate for this study. Symmetric monitoring boundaries are appropriate as one cannot rule out a detrimental relative effect of either strategy of FAST or no-FAST protocols. In a multicenter clinical trial, it is common for early recruitment to be confined to a subset of centers that receive early Institutional Review Board (IRB) approval or have a smoother run-in phase; the experience at these centers may differ from others. Also, a “learning curve” in delivering the study intervention, at some or all centers, is not inconceivable. Because of these issues, we have selected the Lan-DeMets flexible alpha-spending function with O’Brien-Fleming-type boundaries to preserve the overall type I error rate.

The projected accrual period in this study is between 3 and 4 years. The DSMB met prior to study launch and then will meet after 25%, 50%, and 75% of patient accrual. The final analyses will be the fourth evaluation of the data. Thus, there will be three interim analyses, with an additional final analysis of the study data if the study is not terminated early.

### {#20b Methods for additional analyses}

We will additionally measure the proportion of abdominal CT use in the subgroup of children 0 to 3 years old as preliminary data showed the largest difference in this subgroup. This proportion is measured as a categorical variable (yes/no CT scan). We will use the Cochran-Mantel–Haenszel test to determine if differences in CT exist between the two study arms.

### {#20c Methods in analysis to handle protocol nonadherence and any statistical methods to handle missing data}

We anticipate having no missing data for the primary outcomes of abdominal CT and missed/delayed diagnoses of IAIs.

### {#31c Plans to give access to the full protocol, participant-level data, and statistical code}

The study dataset will be made public after planned analyses and manuscripts are completed.

## Oversight and monitoring

### {#5d Composition of the coordinating center and trial steering committee}

The coordinating center consists of the two PIs, research manager, regulatory specialist, data manager, and statistician. The coordinating center meets weekly. The Trial Steering Committee consists of the two PIs, all site PIs, the research manager, all specialty consultants, and the statistician. This committee meets monthly.

### {#21a Composition of the data monitoring committee and its role and reporting structure}

A Data Safety Monitoring Board (DSMB) was established for this study. The composition of this five-member committee includes two separate statisticians, a pediatric emergency medicine physician, a general emergency medicine physician, a pediatric trauma surgeon, and a medical ethicist. The DSMB has approved the initial protocol and will meet after 25%, 50%, and 75% of the sample have been enrolled. The DSMB is independent of the sponsor and reports to the Trial Steering Committee.

### {#22 Adverse event reporting and harms}

All research staff were trained on adverse events and adverse event reporting. This included the possibility of a missed or delayed diagnosis of a child with an IAI. We will systematically assess for complications related to missed/delayed diagnosis of IAIs for all participants. All identified harms will be reported in publications, either in the main manuscript or in online supplements.

### {#23 Frequency and plans for auditing trial conduct}

Remote monitoring visits will be performed at each study site per the study monitoring plan. The purpose of the site monitoring plan is to facilitate site compliance with good clinical practices and Food and Drug Administration guidelines and regulations to verify that the rights and well-being of participants are protected, review any reported adverse events (AE) or serious adverse events (SAEs), and ensure reported data meet attributable, legible, contemporaneous, original, accurate, and complete (ALCOA-C) standards defined as attributable, legible, contemporaneous, original, accurate, complete, consistent, enduring, and verifiable from source documents (source data verification). The trial is also being conducted in compliance with an approved protocol and other applicable regulatory requirements.

At the monitoring visits, the monitor will assess whether the study is being conducted according to the protocol and manual of operations and will educate and advise the participating sites on ways to improve performance. Increased remote or on-site interim monitoring visits may be conducted as necessary based on site performance. The site investigator, lead research coordinator, or other delegated site research staff member will assist with obtaining electronic medical record (EMR) access for all study monitors to allow for source data verification. Any medical records applicable to the study that are received by the site should be available for monitoring as applicable. Site monitoring items are listed in Table 2.

**Table 2:** Site monitoring protocol (reviewed at each interim visit as appropriate)

• Verify receipt of all documents and confirm the latest version of the approved protocol and informed consent is being used.
• Discuss protocol updates and revisions as applicable.
• Discuss enrollment issues and targets.
• Confirm via documentation that informed consent for follow-up contact was obtained for each enrolled participant.
• Verify the most recent version of the case report form (CRF) is being completed and review error correction procedures.
• Confirm the proper participants were enrolled (with particular attention to inclusion/exclusion criteria, randomization procedure, potential adverse events, primary efficacy variables, and good clinical practice compliance) and assess if significant protocol deviations have been made or identified by the site.
• Review regulatory binder for completion.
• Perform reduced source document verification against CRF completion.
• Confirm all required FAST images were uploaded for review.
• Review any reported adverse events or serious adverse events (AE/SAEs).
• Review reporting of significant protocol deviations to the IRB within the appropriate timelines.
• Review personnel changes and their training documentation. All Research Coordinators who joined the study after site initiation on 02 February 2023 must complete a Research Coordinator Training Checklist
• Any other issues as deemed important to the conduct of the study.

### {#25 Plans for communicating important protocol amendments to relevant parties}

Any significant changes to the protocol will be shared with all sites and their respective personnel as well as with the DSMB.

### {#31a Dissemination plans}

Trial results will be disseminated via multiple methods. This includes presentations at national research meeting(s) and submissions to journal(s) for publication.

## Discussion

The United States of America (USA) population has seen a sevenfold increase in medical imaging radiation exposure over the last 30 years [40]. Although only 10–15% of all radiographic studies in the USA are CT, these account for most (67–75%) of the collective radiation from diagnostic imaging [14, 41]. Radiation exposure from CT has several adverse effects in children beyond radiation-induced malignancies, including decreased cognitive function [12, 14, 16, 19, 42, 43]. Efforts to decrease CT use in children have had some recent success as the rapid increase in use has plateaued [44]. However, CT use in injured children remains excessive [45].

Children are more susceptible than adults to developing malignancies from diagnostic radiation [13, 15]. This is due to the increased sensitivity to radiation of developing pediatric organs, as well as longer life expectancy after exposure, thereby increasing the latency period in which adverse effects can be expressed. The current estimates of lifetime attributable risk of cancer from one current-generation abdominal CT range from one Malignancy per 450 scans for infants to one per 570 for children 15 years old. Although these are averages, the risks for girls are nearly double that for boys, and this risk is five times that of adults [19].

Due to the lack of precise evidence regarding indications for trauma CT use in children, CT use varies greatly by center [46]. Abdominal CT use varies substantially even between academic centers (from 9 to 68% of enrolled patients in a PECARN torso trauma study) [8]. Substantial increase in abdominal CT has occurred; however, the increased use of CT has not improved clinical outcomes [47–51]. Prediction rules exist for the use of abdominal CT, although evaluation strategies when patients are positive for the rule are unclear [52].

The FAST examination was originally developed for trauma assessment in adults to identify hemoperitoneum. Intraperitoneal fluid (i.e., blood) after trauma is detected as an area with anechogenicity indicative of fluid or heterogeneous complex attenuation due to the presence of hemorrhage (Fig. 1). The FAST examination for detection of hemoperitoneum in trauma consists of several anatomic views. These include a right hepatorenal interface (Morison’s pouch) view, perisplenic view, and longitudinal and transverse views of the pelvis.

Controversies and variations in using the FAST examination in pediatric trauma were highlighted in a survey of general emergency physicians, pediatric emergency physicians, and trauma surgeons [53]. In the case of adult trauma patients, 91% of the respondents considered the FAST to be “somewhat to extremely useful.” By contrast, in the case of pediatric trauma, 73% of all respondents, but only 57% of pediatric emergency physicians, considered the FAST examination to be useful [53]. In that survey, the authors recommended further research to evaluate the clinical utility of the FAST examination for pediatric trauma patients.

Whereas randomized controlled trials exist for the FAST in injured adults [25, 54], limited data exist in children. FAST performance may also be different in children than adults as locations of intraperitoneal fluid collection are different in children [55]. In uncontrolled studies, the sensitivity of the FAST for hemoperitoneum in injured children has been good, especially in the most severely injured/hypotensive children [32, 56]. Although both abdominal CT and the FAST have excellent specificities, systematic reviews indicate the sensitivity of the FAST for hemoperitoneum in children ranges from 35% to 80%, much lower than CT [30, 56]. This suggests that an ED evaluation protocol using the FAST in children may have clinical utility in some pediatric trauma patients but requires investigation.

In a 2007 systematic review, the negative likelihood ratio for the FAST examination suggests that patients at low risk for IAI may benefit from screening with the FAST examination, as a negative FAST in this setting has a very low posterior probability for IAI [56]. A recent systematic review and meta-analysis had similar conclusions [30]. Although the FAST cannot exclude IAI in all injured children due to its limited sensitivity, it may be sufficient to avoid CT in children at low risk for IAI [30].

A large, multicenter observational study in children with blunt abdominal trauma led by the study investigators demonstrated that FAST use at participating sites was highly variable (from 0.5% to 88% of patients) [31]. Use of the FAST, however, was associated with decreased CT use in those children considered to be at 1–5% or 6–10% risk of IAI prior to the FAST (relative risk of obtaining a CT compared to those without the FAST examination performed was 0.81 and 0.85, respectively) [31]. However, in a single-center randomized controlled trial of the FAST conducted by the study investigators, there was no difference in abdominal CT use in those who were randomized to the FAST versus no-FAST study arms [28]. Clinician suspicion of IAI, however, was significantly decreased in the FAST arm following FAST examination [28]. The decrease in clinician suspicion without a corresponding decrease in CT use in the FAST study arm may be a result of clinician awareness of the imperfect sensitivity of the FAST, particularly in injured children, or insufficient confidence in interpreting the images. It may also be a result of modifiable factors (training, confidence, etc.) that if intervened upon may safely reduce CT use.

We considered a cluster-randomized design at the level of the clinician, which could minimize the risk of intervention nonadherence. However, based on prior experience in our single-center trial in which crossover was rare, we anticipated low rates of nonadherence. Given the additional complexity of a cluster randomized trial, we determined that patient-level randomization remained the most efficient and scientifically rigorous approach. We acknowledge this as a potential limitation but expect a minimal impact on study integrity.

Missed or delayed diagnoses of IAIs are anticipated to be rare. Therefore, a feasible sample size to determine clinically important differences in the two arms in this outcome was not feasible. We chose to include it as a primary outcome, however, because clinicians need to be assured that use of FAST does not increase the frequency of missed IAIs or delay the diagnosis.

Finally, although beyond the primary scope of this manuscript, participating clinicians will complete a baseline survey at the start of their participation. Together with trial data, this survey will be used to explore patient, physician, and system-level factors associated with obtaining abdominal CT scans in patients deemed low risk for intra-abdominal injury following a negative FAST examination. This sub-analysis will be conducted within the cohort randomized to the FAST arm.

As there are insufficient data to determine whether a strategy employing the FAST examination in the evaluation of hemodynamically stable injured children decreases abdominal CT use, it improves patient safety and improves clinical efficiency (shortened time to diagnosis or disposition). The current study addresses the limitations of previous studies and will definitively answer the question regarding the utility of the FAST in hemodynamically stable injured children. In summary, this randomized controlled trial will evaluate a strategy of routine FAST examination in the evaluation of children with blunt abdominal trauma. Additional data from this study will identify modifiable factors associated with the use of CT scans in low-risk children with normal FAST examinations. These data will improve care for injured children by reducing variability, promoting safety and quality, and limiting exposure to unnecessary CT scans and associated radiation.

## Trial status

The trial is currently enrolling participants. The first patient is enrolled on May 3, 2023. The trial is expected to enroll participants through May 2027 (Fig. 4).

## Supplementary Information

Additional file 1. Appendix: {#32 Informed consent materials}. Appendix item 1: Patient/Guardian Information Sheet. Appendix item 2: Consent documents. {#33 Biological specimens}. No biological data, including laboratory values or biospecimens outside routine clinical care, are being collected as part of this trial.

## Data Availability

All investigators will have access to a deidentified database after study completion.

## Abbreviations

AE: adverse events
ALCOA: Attributable, Legible, Contemporaneous, Original, Accurate, and Complete
CI: confidence intervals
CT: computed tomography
DSMB: data safety monitoring board
ED: Emergency Department
EMR: electronic medical record
FAST: focused assessment with sonography for trauma
IAI: intra-abdominal injury
IRB: Institutional review board
PECARN: Pediatric Emergency Care Applied Research Network
PI: Principal Investigator
REDCap: Research Electronic Data Capture
SAS: Statistical Analysis System
SAE: serious adverse events
USA: United States of America
