# Supplementary material for: Abdominal ultrasound (FAST) in hemodynamically stable children with blunt abdominal trauma: study protocol for a randomized controlled trial
Source: Trials. 2025 Dec 12;26:564. doi: 10.1186/s13063-025-09137-6 (PMC12699813; doi:10.1186/s13063-025-09137-6)
Supplement: Supplementary file 1 — Additional file 1. Appendix: {#32 Informed consent materials}. Appendix item 1: Patient/Guardian Information Sheet. Appendix item 2: Consent documents. {#33 Biological specimens}. No biological data, including laboratory values or biospecimens outside routine clinical care, are being collected as part of this trial. [file 13063_2025_9137_MOESM1_ESM.pdf]

Information Sheet

Research Study Title: A randomized controlled trial of abdominal ultrasound (**F**ocused **A**ssessment with **S**onogram for **T**rauma) in children with blunt torso trauma
Principal Investigators: James Holmes, MD, MPH & Nathan Kuppermann, MD, MPH

Introduction and Purpose

The investigators listed above are conducting a research study on children younger than 18 years who arrive at the Emergency Room with abdominal trauma (injured or hurt belly). The goal of the research is to better understand the best way to treat these types of injuries. Abdominal CT or CAT scan (type of x-ray of the abdomen/belly) is the usual test used to evaluate for abdominal trauma. Abdominal CT scans, however, have drawbacks including the time to complete the CT scan and significant radiation exposure to the patient. The purpose of this study is to determine if using an abdominal ultrasound can help in the evaluation of children with abdominal trauma by decreasing the patient’s wait time for evaluation and exposure to unnecessary CT scans. The FAST exam is an abdominal ultrasound, which is a quick method to evaluate injured patients for bleeding inside the abdomen. It can be performed at the patient’s bedside within 1-5 minutes and uses no radiation. We are conducting this study to learn if abdominal ultrasound should be part of the initial evaluation of children with abdominal trauma to more quickly assess and decrease radiation exposure from CT scans.

Your child was recently seen for abdominal trauma and we would like to ask some questions about their outcome after leaving the Emergency Room. To collect the information, we will ask you to complete a brief text or telephone survey within 3 months of leaving the Emergency Room. The survey should take approximately 15 minutes or less. Some of these questions might make you feel uncomfortable or upset. You do not have to answer any of the questions you do not want to answer. If you agree, we will use the information you provide, combined with information from your child’s medical record, to help understand if a FAST exam can help doctors evaluate children with abdominal trauma more quickly and with fewer CT scans.

Here are some issues to think about before you decide whether to participate in this research:
Participating in research is completely voluntary. You can choose to take part now and change your mind in the future. Whatever you decide, there will be no penalty to you or loss of benefits to which you or your child is otherwise entitled. Your child’s care will otherwise be exactly the same.

You or your child will not receive any direct benefit if you take part in this study. We hope that information obtained from the study will improve care of injured children in the future.

Confidentiality

As with all research, there is a chance that confidentiality could be compromised. To minimize the risks of loss of confidentiality, we will not include any information that directly identifies your child on the information we collect, or on the information resulting from the research. Instead, we will record a code on the data, and we will keep a link between the code and your child’s identity in a different location.

Do not write below this line. For IRB stamp and version date only.

APPROVED by the Institutional Review Board at the University of California, Davis	
Protocol	APPROVED
1943799	April 3, 2023

If your child is treated at a different hospital, you may be asked to sign a separate form to give your permission for research staff to work with that hospital to review your child's medical records. Your child's medical records may become part of the research record. If that happens, your child's medical records may be looked at by this institution, the sponsor of this study, the Data Safety Monitoring Board, the Institutional Review Board, and government agencies or other groups associated with the study. They may not copy or take your child's personal health information from their medical records unless permitted or required by law.

We will use your child's information to conduct this study. Information collected for this research may also be used for future research studies. We will not share any personally identifiable information. Our goal is to make more research possible. These studies may be done by researchers at this institution or other institutions, including commercial entities. Information may be placed in one or more external scientific databases for access and use. We will not ask you for additional permission to share de-identified information.

This research is covered by a Certificate of Confidentiality (CoC) from the National Institutes of Health. The researchers with this CoC may not disclose or use information, documents, or biospecimens that may identify your child in any federal, state, or local civil, criminal, administrative, legislative, or other action, suit, or proceeding. For example, the information collected in this research cannot be used as evidence in a proceeding unless you consent to this use. Information, documents, or biospecimens protected by this CoC cannot be disclosed to anyone else who is not connected with the research, except:

- To a federal agency sponsoring this research when information is needed for auditing or program evaluations;
- To meet the requirements of the U.S. FDA;
- If a federal, state or local law requires disclosure such as a requirement to report a communicable disease;
- If information about you or your child must be disclosed to prevent serious harm to yourself, your child, or others such as child abuse, elder abuse or spousal abuse;
- If you consent to the disclosure, including for your child's medical treatment, to an insurer or employer to obtain information about your child; or
- If it is used for other scientific research, as allowed by federal regulations protecting research subjects.

This CoC also does not prevent you or a family member from voluntarily releasing information about yourself or your child and your involvement in this research.

Questions

If you have any questions about this research, please feel free to contact the investigator James F. Holmes, MD, MPH at 916-734-1533 or jfholmes@ucdavis.edu.

If you have any questions about your rights or treatment as a research participant in this study, please contact the single Institutional Review Board (IRB) of record, UC Davis IRB by phone: 916-703-9158 or by email: HS-IRBEducation@ucdavis.edu. Thank you for your help in this important study!

Do not write below this line. For IRB stamp and version date only.

APPROVED by the Institutional Review Board at the University of California, Davis	
Protocol	APPROVED
1943799	April 3, 2023
